# Characterization of a New Chronic Lymphocytic Leukemia Cell Line for Mechanistic *In Vitro* and *In Vivo* Studies Relevant to Disease

**DOI:** 10.1371/journal.pone.0076607

**Published:** 2013-10-09

**Authors:** Erin Hertlein, Kyle A. Beckwith, Gerard Lozanski, Timothy L. Chen, William H. Towns, Amy J. Johnson, Amy Lehman, Amy S. Ruppert, Brad Bolon, Leslie Andritsos, Arletta Lozanski, Laura Rassenti, Weiqiang Zhao, Tiina M. Jarvinen, Leigha Senter, Carlo M. Croce, David E. Symer, Albert de la Chapelle, Nyla A. Heerema, John C. Byrd

**Affiliations:** 1 Department of Internal Medicine, Division of Hematology, Comprehensive Cancer Center at The Ohio State University, Columbus, Ohio, United States of America; 2 Department of Pathology, the Ohio State University, Columbus, Ohio, United States of America; 3 Center for Biostatistics, the Ohio State University, Columbus, Ohio, United States of America; 4 Department of Veterinary Biosciences and the Comparative Pathology and Mouse Phenotyping Shared Resource, the Ohio State University, Columbus, Ohio, United States of America; 5 Moores University of California-San Diego Cancer Center, University of California San Diego, California, United States of America; 6 Department of Molecular Virology, Immunology and Medical Genetics, Division of Human Cancer Genetics, Comprehensive Cancer Center at the Ohio State University, Columbus, Ohio, United States of America; University of Manitoba, Canada

## Abstract

Studies of chronic lymphocytic leukemia (CLL) have yielded substantial progress, however a lack of immortalized cell lines representative of the primary disease has hampered a full understanding of disease pathogenesis and development of new treatments. Here we describe a novel CLL cell line (OSU-CLL) generated by EBV transformation, which displays a similar cytogenetic and immunophenotype observed in the patient’s CLL (CD5 positive with trisomy 12 and 19). A companion cell line was also generated from the same patient (OSU-NB). This cell line lacked typical CLL characteristics, and is likely derived from the patient’s normal B cells. *In vitro* migration assays demonstrated that OSU-CLL exhibits migratory properties similar to primary CLL cells whereas OSU-NB has significantly reduced ability to migrate spontaneously or towards chemokine. Microarray analysis demonstrated distinct gene expression patterns in the two cell lines, including genes on chromosomes 12 and 19, which is consistent with the cytogenetic profile in this cell line. Finally, OSU-CLL was readily transplantable into NOG mice, producing uniform engraftment by three weeks with leukemic cells detectable in the peripheral blood spleen and bone marrow. These studies describe a new CLL cell line that extends currently available models to study gene function in this disease.

## Introduction

Chronic lymphocytic leukemia (CLL) is one of the most common types of adult leukemia, and is characterized by a typical immunophenotype of CD19, CD20, CD23 with co-expression of the pan T-cell marker CD5. Our knowledge of CLL biology has expanded dramatically with recognition of two disease subsets which are categorized by the mutational status of the immunoglobulin heavy chain variable region (IGHV). Patients with mutated IGHV typically have more indolent disease, low risk genetic aberrations, lack high risk gene mutations (p53, NOTCH-1, and SF3B1) and exhibit minimal clonal evolution over time. In contrast, patients with un-mutated IGHV disease have an increased frequency of high-risk genomic features, genetic mutations, and exhibit clonal evolution [1–7]. In this latter patient group, adverse outcomes are associated with over-expression of ZAP-70, which may enhance BCR signaling and migration toward chemokine and stromal cells [8]. These biologic features translate to clinically meaningful differences between these two CLL types, where patients with mutated IGHV have a longer treatment-free survival interval, and improved progression-free and overall survival compared to patients with un-mutated IGHV [9,10].

Despite these biological and clinical differences, both groups share common features such as differential expression of certain mRNAs and miRNAs when compared to normal B-cells [11–14]. Attempts to study the effects of differentially expressed genes in CLL are complicated by the high spontaneous apoptosis rate of cultured tumor cells. Additionally, primary CLL cells are quite difficult to transfect with expression vectors or siRNA constructs and lack the ability to expand or recapitulate disease features when engrafted into immunodeficient mice. Unlike many other hematopoietic malignancies, the limited number of CLL cell lines has impeded rigorous mechanistic interrogation of both coding and non-coding gene function, regulation, and interaction with other genes, as well as response to CLL therapeutic agents [15].

The resistance of primary CLL cells to viral transformation by Epstein-Barr virus (EBV) has been cited as a major reason for this lack representative immortalized cell lines which accurately reflect the disease [16–18]. The few existing CLL lines have molecular features of high-risk, IGHV un-mutated CLL and lack many primary features associated with clinical CLL. The most well characterized CLL cell line, MEC1, bears mutated *TP53* as well [19]. Attempts to expand the number of available CLL lines have been reported. CLL cells may be maintained in culture following EBV transformation using cell feeder layers or other B-cell activation stimuli; however, over time in culture these cells may exhibit diminished CD5 expression [20,21]. However these difficulties may be overcome by developing hetero-hybridoma cell lines to create stable *in vitro* cultures from CLL patient samples [22]. CLL lines with IGHV mutated disease are not widely available (one previous study describes a CD5+ cell line with mutated IGHV) [21]. Herein, we describe an EBV-transformed CLL cell line with mutated IGHV, trisomy 12, trisomy 19, non-complex karyotype and wild type p53. This novel cell line displays an immunophenotype similar to human CLL, remains stable following extended culture, is readily manipulated by stable gene transfection, and is reproducibly engrafted into immunodeficient mice. As such, the OSU-CLL cell line provides a unique tool to rigorously study the biology of CLL.

## Methods

### Ethics Statement

Blood was obtained from CLL patients after obtaining written, informed consent according to an Ohio State University Institutional Review Board (IRB) approved protocol, in agreement with the principles of the Declaration of Helsinki. This IRB approved protocol stated that collected samples will be used for the following purposes: To establish a tissue repository of blood, genomic DNA, fibroblast and lymphoblastoid cell lines from CLL patients. All animal research was reviewed and approved by The Ohio State University Institutional Animal Care and Use Committee.

### EBV Immortalization

Peripheral blood mononuclear cells isolated from the patient were infected with the B95-8 strain of EBV virus in the presence of cyclosporin A. Cells were expanded and cryo-preserved when the outgrowth of the EBV was evident based on large clusters of cells. Cell lines were maintained in RPMI media supplemented with 10% fetal bovine serum and antibiotics.

### Immunophenotyping

Cell lines were analyzed for a panel of CLL surface markers using a five color technique with a gating strategy based on CD45 and side scatter characteristics. Data was analyzed on a FC500 flow cytometry analyzer equipped with CXP software vs 2.2 and prism plot utility (Beckman Coulter, Miami FL, USA). Detailed methods and antibodies utilized are described in supplemental material.

### Viability Assays

Cell viability was determined by MTS (3-(4,5-dimethylthiazol-2-yl)-5-(3-carboxymethoxyphenyl)-2-(4-sulfophenyl)-2H-tetrazolium) assay or staining with Annexin V-FITC and propidium iodide (PI).

### Fluorescence In Situ Hybridization (FISH) and Karyotype

CpG stimulated karyotype and FISH were performed as previously described [23].

### Immunoglobulin Gene Mutational Analysis

DNA from each sample is amplified using IGHV family-specific primers for the sense strand and antisense JH degenerate primer. The PCR products are size selected by electrophoresis in 2% agarose and sequenced directly [24,25]. Nucleotide sequences are analyzed using the ImMunoGenetic (IMGT) directory [26]. Somatic mutations are identified by comparison with the most homologous germline IGHV gene. The mutational status is determined by dividing the number of nucleotide differences between the 5' end of framework 1 (FR1) and the 3' end of FR3 by the number of IGHV nucleotides. Sequences with less than 98% homology with the corresponding germline IGHV gene are considered mutated.

### Gene Mutational Analysis

Genomic DNA was extracted from 1x10^7^ cells. All regions of interest were amplified with primers designed to cover either entire exons, or specific exons or SNPs which have been previously identified [27–29]. Primer sequences, PCR amplification conditions and detailed description methods are described in supplemental material. Temperature gradient capillary electrophoresis (TGCE) was performed as previously described [30], and samples with abnormal peaks were validated by bidirectional Sanger sequencing.

### Immunoblot Analysis

Immunoblots were performed as described [31]. Antibodies used included EBV proteins LMP1, EBNA2, EBNA3a and BZLF-1 and Actin (Santa Cruz Biotechnology, Santa Cruz CA).

### Migration Assays

Cells were suspended in RPMI at 5x10^6^ cells/mL, and 100 µl was placed in the upper chamber of a 24-transwell plate with a 5µm filter. Chambers were placed into wells containing media containing no chemokine (control), recombinant human CXCL12 (200ng/mL) or CXCL13 (1000ng/mL). Migration was permitted for 3 hours, and cells in the lower chamber were collected and counted for 20 seconds on high speed on a Beckman Coulter FC500 flow cytometer. A 1/20 dilution of input cells was also determined.

### Animal Studies

A total of ten NOG mice (Taconic, Cambridge City, IN) were engrafted with 1x10^7^ OSU-CLL cells by lateral tail vein injection. Mice were sacrificed upon development of hind limb paralysis, at which point peripheral blood, spleen and bone marrow were collected and analyzed for the presence of CD19/CD5 positive cells. White blood cell count is determined by modified Giemsa stain (Fisher Scientific, Pittsburg, PA). All experiments were carried out under protocols approved by the OSU Institutional Animal Care and Use Committee.

### Statistical Analysis

For the *in vitro* migration assays, a mixed effects model was applied to the overall migration (% of input, log-transformed), and the migration towards CXCL12 and CXCL13 relative to control was compared between the OSU-CLL and OSU-NB cell lines using an interaction contrast. Similarly, differences between the therapeutic antibodies + a-FC crosslinker vs. untreated were estimated, with 95% confidence intervals (CI) from a mixed effects model. P-values were adjusted using Holm’s method to control the family-wise error rate at 0.05. Four-parameter logistic regression models were used to calculate IC_50_ values of drug treatments, where possible [32]. All analyses were performed using SAS/STAT software, v9.2 (SAS Institute, Inc., Cary, NC).

### Microarray Analysis

RNA samples from the cell lines were analyzed for differential expression using Affymetrix U133 plus 2.0 GeneChips (Affymetrix, Santa Clara, CA). Briefly, summary measures of gene expression were computed using the robust multichip average method, which utilizes quantile normalization across arrays. A filtering step was performed to remove probe sets with expression values <100 in both the OSU-CLL and OSU-NB cell lines. Fold changes were calculated as the ratio of expression in one cell line relative to the other. The top 50 genes over- and under-expressed (2-fold cut-off) are described.

## Results

### Establishment of CLL-like Line

**Figure 1:**
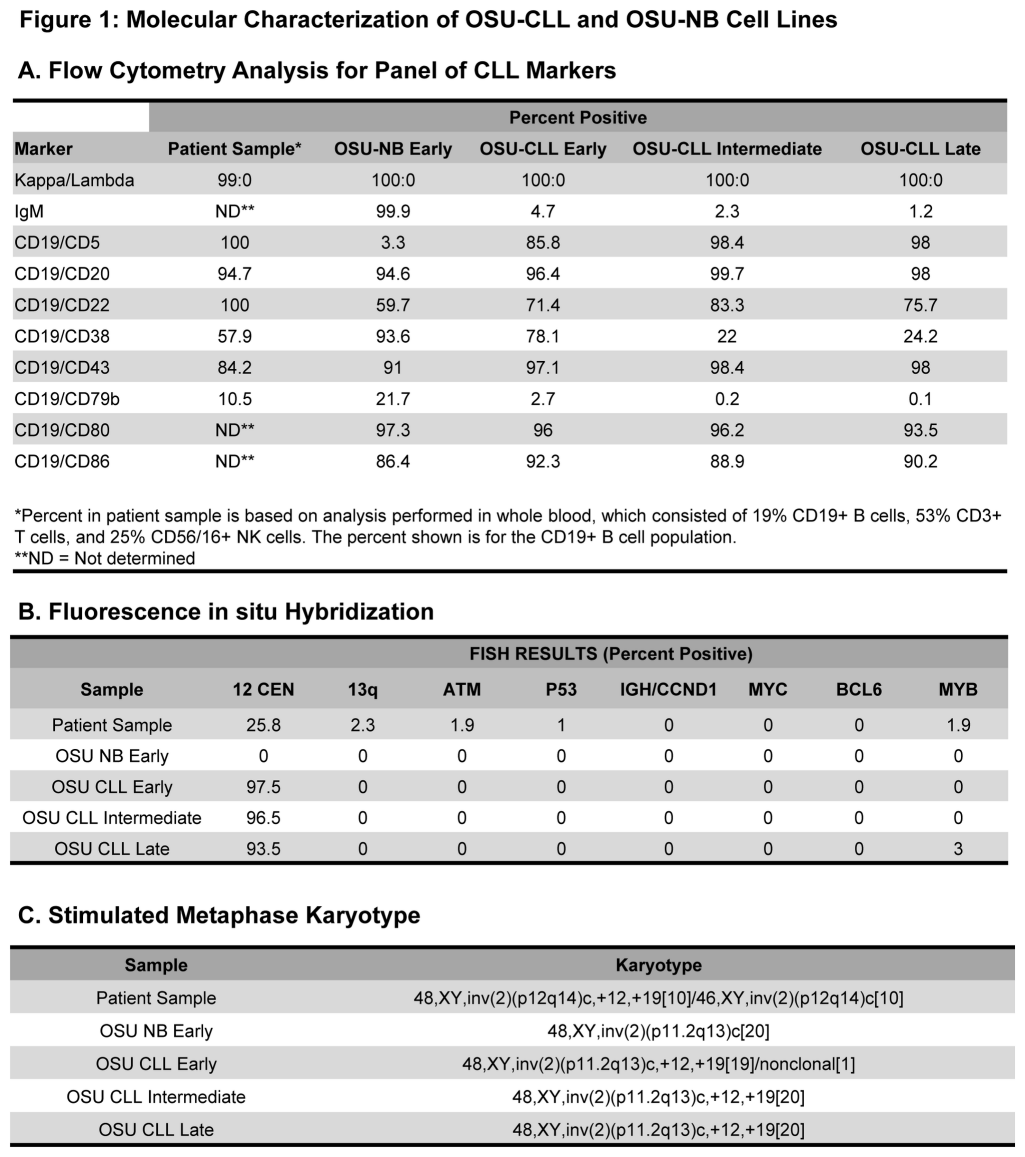
Molecular characterization of OSU-CLL and OSU-NB cell lines. Analysis is performed in patient sample from which the cell lines were derived as well as cultured cells from both lines (approximate timing: early = 3 months, intermediate = 6 months, late = 9 months). **A**. **Flow cytometric analysis for CLL related surface molecules**. Flow cytometric analysis for selected B-cell markers, shown as the percent of cells positive. **B**. **Fluorescence in situ hybridization**. Interphase FISH analysis for a panel of cytogenetic abnormalities associated with CLL: trisomy 12 (centromere), del(13q14) (D13S319), del(11q22.3) (ATM), del(17p13.1) (TP53), t(11;14) (CCND1-IGH fusion), +8 (MYC), +3 (BCL6), and del(6q22.3) (c-MYB). **C**. **Karyotype analysis**. Metaphase karyotype analysis to determine any additional chromosomal abnormalities not identified by FISH analysis.

Establishing a lasting source of germ line material for genetic studies in CLL is challenging due to the disseminated nature of the disease. Previous studies have shown primary CLL cells are typically resistant to EBV transformation whereas normal B lymphocytes are not [17,18], thus prompting exploration of *ex vivo* EBV transformation of PBMCs isolated from CLL patients as a source of germ line DNA derived from normal B-cells. A total of 21 patient PBMCs underwent EBV transformation, of which 20 derived a CD19, CD20, CD22, CD79b positive cell line lacking CD5. Unexpectedly, one line yielded a CLL-like clone (OSU-CLL) whose immunophenotype differed dramatically, as it co-expressed CD5, a pan-T cell marker typically seen in CLL patient samples. However, a companion cell line (OSU-NB) generated from PBMCs from the same patient sample five years after derivation of the original line, resembles a normal EBV-infected B-cell and is CD5 negative. Both cell lines grow in large clumps which is characteristic of EBV-infected B-cells (Figure S1A). Immunoblot analysis indicates that both cell lines express multiple EBV markers, which were maintained during early, intermediate and late passages (3, 6, and 9 months in culture, respectively) indicating that they each were successfully virally transformed (Figure S1B). These two cell lines exhibit differential expression of surface IgM, CD22, and CD38, which remains stable over time in culture, with the exception of CD79b and CD38, which decrease in the OSU-CLL line after extended time in cell culture (Figure 1A). Both cell lines maintained high expression of CD80 and CD86, likely due to activation by EBV. The potential for these cell lines to be utilized for *in vivo* and *in vitro* analysis of molecular differences in CLL therefore prompted detailed characterization of OSU-CLL and OSU-NB.

### OSU-CLL Line Has Genetic Features Similar to the Founder Patient’s CLL

**Figure 2:**
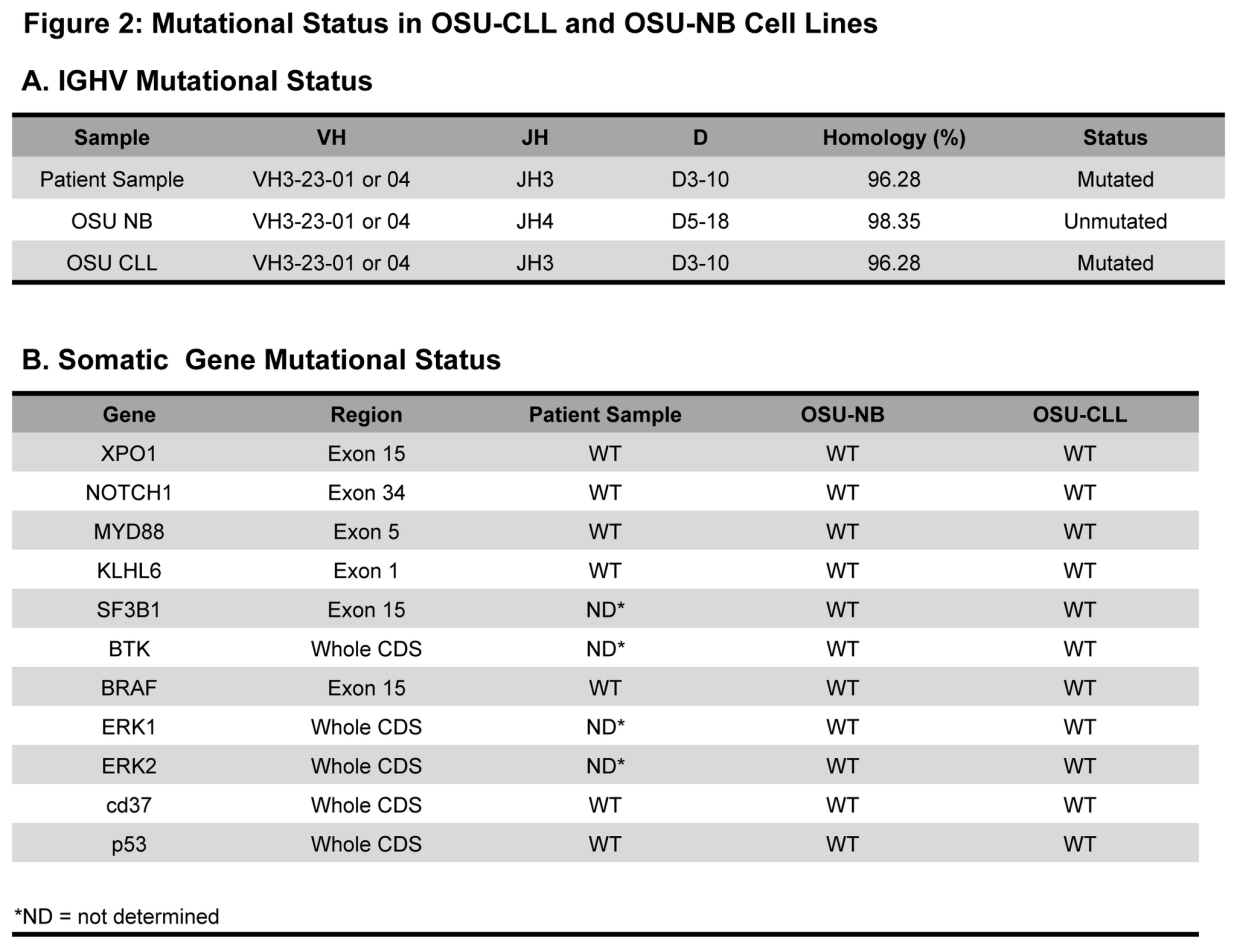
Mutational status in OSU-CLL and OSU-NB cell lines. Analysis is performed in patient sample from which the cell lines were derived as well as cultured cells from both lines (approximate timing: early = 3 months, intermediate = 6 months, late = 9 months). **A**. **IGHV mutational status**. Gene mutational status (relative to the reference genome), and immunoglobulin gene usage determined by sequence analysis. **B**. **Somatic gene mutation status**. Existence of mutations for common CLL variants was explored by sequence analysis.

OSU-CLL was derived from a previously treated CLL patient. The patient’s metaphase karyotype when OSU-CLL was generated was 48, XY, +12, +19. The patient also displayed an inversion of chromosome 2. Interphase cytogenetic studies for del(13q14) (D13S319), del(6q22.3) (c-MYB), del(11q22.3) (ATM), del(17p13.1) (TP53), +3 (BCL6), +8 (MYC), +12 (centromere) and t(11;14) (CCND1-IGH fusion) revealed no additional genomic lesions common in CLL. Parallel metaphase karyotype and interphase cytogenetic analysis in the cell lines demonstrated OSU-NB is cytogenetically normal (except for the chromosome 2 inversion), whereas OSU-CLL mirrored findings in the CLL patient (Figure 1B and 1C). This cytogenetic profile is particularly interesting given that the co-association of trisomy 12 and trisomy 19 is relatively rare in CLL. To further determine if OSU-CLL was derived from the same clone as the patient’s CLL, we examined the heavy chain mutational status. The patient sample as well as both cell lines has a VH rearrangement of 3-23. However, OSU-CLL (but not OSU-NB) also shares the same JH and D rearrangement and mutated IGHV status present in the patient sample (Figure 2A). This further confirms that OSU-CLL is derived from the patient’s CLL clone, whereas OSU-NB is derived from a normal B cell. Finally, we determined the status of several common sequence variants observed in CLL (p53, XPO1, Notch-1, MyD88, KLHL6, ERK1, ERK2, BTK, CD37, and SF3B1) using mutational screening. We found that these mutations were absent in the patient’s primary CLL cells as well as both cell lines (Figure 2B). The differences between these two cell lines on an otherwise identical genetic background provide a very unique opportunity to study CLL biology.

### OSU-CLL Demonstrates Stable Growth and Genetic Properties after Prolonged Culture

OSU-CLL has been maintained independently of feeder layers or additional growth factors for the period of one year. The growth rate of OSU-CLL is consistent with other reported lymphoblastoid lines, with a doubling time of approximately 50 hours during early passages which increases slightly as the cells are cultured over longer periods of time. Serial assessment of the immunophenotype, karyotype and interphase cytogenetics during this time demonstrated no significant change in OSU-CLL, with the exception of the previously mentioned decrease in CD79b and CD38 expression (Figure 1). In contrast, the doubling time for OSU-NB is approximately 30 hours early on, gradually slowing until cells undergo apoptosis (after approximately 35 *in vitro* passages).

### OSU-CLL Line Gene Expression Profile Accurately Represents Primary CLL

The mRNA expression levels in the two cell lines were compared to those reported for normal B-cells and CLL B-cells. The top 50 over- and under-expressed genes by at least 2-fold are shown in Tables S1 and S2, respectively. Several genes known to be deregulated in CLL are similarly recapitulated in OSU-CLL relative to OSU-NB, such as CD5, LEF1 [33,34], CXCR4 [35], and BAG3 [36]. In addition, given that OSU-CLL has an extra copy of chromosomes 12 and 19, we hypothesized that increased expression of genes on these chromosomes would be over-represented in OSU-CLL, as this has been demonstrated in primary CLL with trisomy 12 [37]. We found that 7.9% of genes on chromosome 12 and 4.3% of genes on chromosome 19 are up-regulated in OSU-CLL relative to OSU-NB, compared to 2-3% of genes over-expressed on other chromosomes (Figure S2).

### OSU-CLL Line Exhibits Differential *In Vitro* Migration Properties

**Figure 3:**
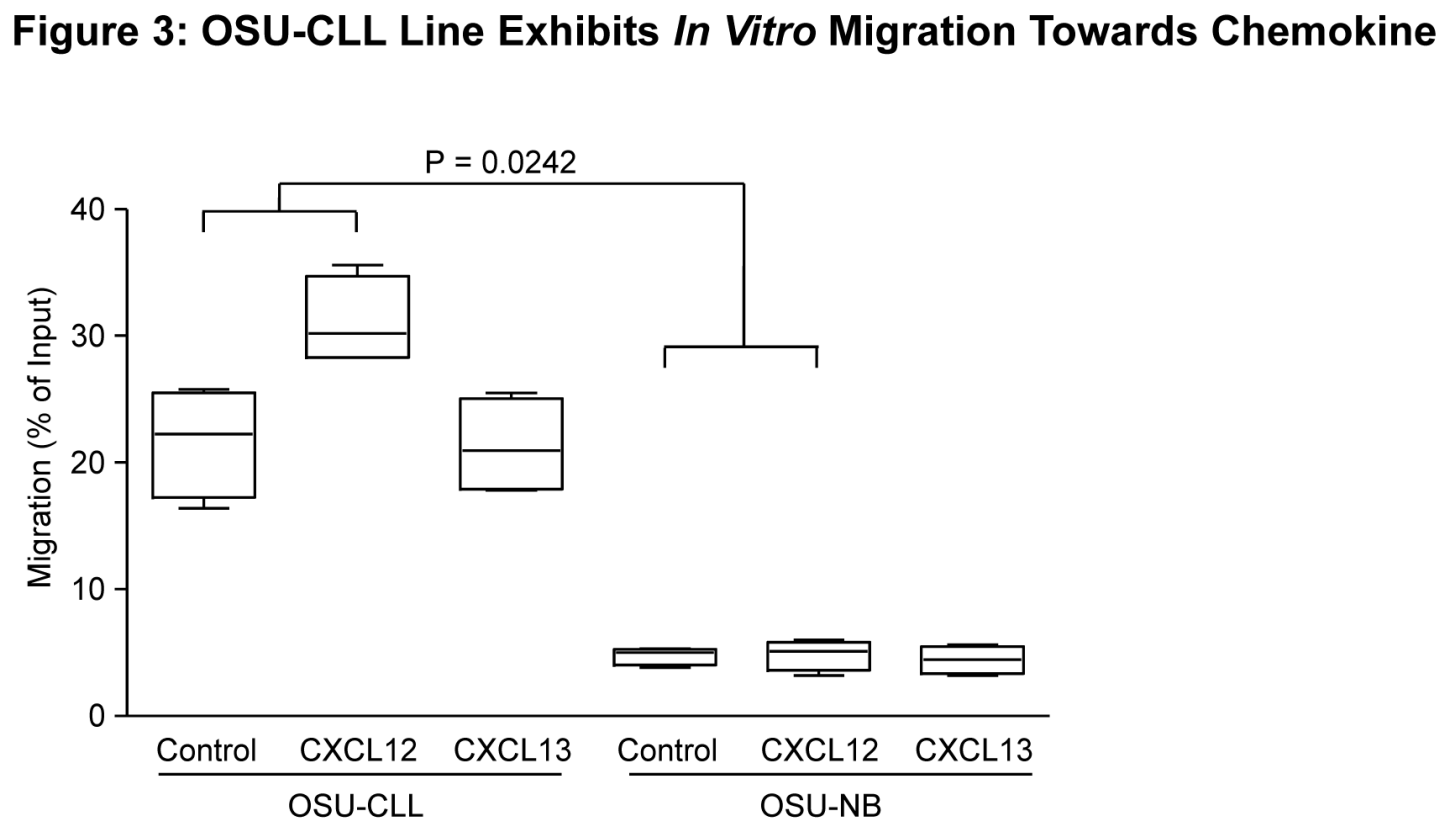
OSU-CLL and OSU-NB exhibit differential *in vitro* migration properties towards chemokine. Cells were suspended (5 x 10^6^ cells/mL) and placed in the upper well of 24-well transwell plates. The bottom wells contained either media alone, or media with recombinant CXCL12 (200 ng/mL) or CXCL13 (1000 ng/mL). Cells in the lower chamber were collected after 3 hours; percent migration is calculated relative to the input.

Homing of tumor cells to protective niches in the microenvironment is accomplished by recruiting CLL cells via interaction of cell surface receptors with chemokines produced from stromal cells. *In vitro* migration assays indicated that OSU-CLL cells exhibits greater migration towards recombinant CXCL12 than OSU-NB (P = 0.02) (Figure 3). These migratory properties are similar to those reported for primary CLL cells [38], and likely is related to differential expression of CXCR4, the receptor for CXCL12 (Table S1). Interestingly, the migration of OSU-CLL towards media containing no chemokine (control) was also significantly greater than that of OSU-NB (P < 0.0001), indicating that intrinsic differences between cell lines mediate cell motility independent of chemokine or chemokine receptor levels. Neither line demonstrated any significant migration (relative to the control media) toward CXCL13. These results indicate that OSU-CLL and OSU-NB are valuable tools to study cell migration and signaling from the microenvironment in CLL, which contributes to CLL cell resistance to apoptosis.

### OSU-CLL Responds to CLL Relevant Therapies

**Figure 4:**
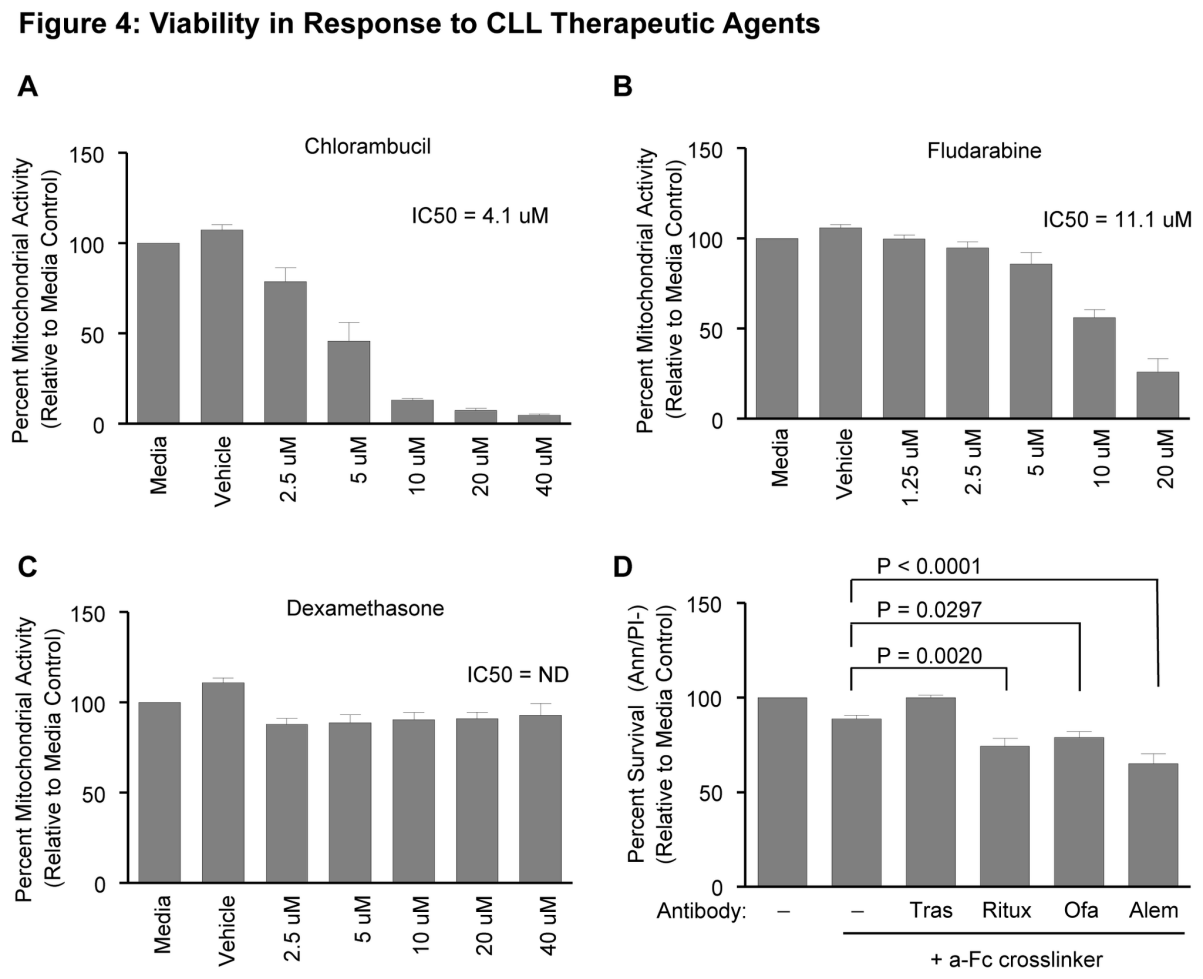
Viability OSU-CLL in response to CLL therapeutic agents. **A**. Viability of OSU-CLL either untreated (media), treated with vehicle control (DMSO), or increasing doses of chlorambucil (**A**), fludarabine (**B**) and dexamethasone (**C**) for 48 hours determined by MTS (3-(4,5-dimethylthiazol-2-yl)-5-(3-carboxymethoxyphenyl)-2-(4-sulfophenyl)-2H-tetrazolium) assay. **D**. Viability at 48 hours in response to the indicated therapeutic antibodies was determined by AnnexinV/ propidium iodide (Ann/PI) staining. Antibodies were used as a concentration of 10 µg/mL in the presence of 50 µg/mL anti-Fc crosslinking antibody. Abbreviations: Trastuzumab (Tras, HER2), Rituximab and Ofatumumab (Ritux and Ofa, CD20) and Alemtuzumab, (Alem, CD52). All results shown are representative of 4 independent experiments.

Cell lines are utilized in part to screen therapeutic agents relevant to the therapy of the respective disease. In this regard, we evaluated the effectiveness of several different therapeutic agents currently approved for the treatment of CLL using OSU-CLL (passage 50). While OSU-CLL responds to chlorambucil treatment (48 hour IC_50_ = 4.1) (Figure 4A), there was only a significant IC_50_ reached with a super-physiological dose of fludarabine (48 hour IC_50_ = 11.1) (Figure 4B) and no significant IC_50_ reached with dexamethasone (Figure 4C). In addition to chemotherapeutic agents, we also tested the ability of OSU-CLL to respond to biological therapeutic antibodies. OSU-CLL shows significant response to 48 hour treatment with rituximab (63.8% vs. 53.4%; P = 0.0020), ofatumumab (63.8% vs. 56.6%; P = 0.0297) and alemtuzumab (63.8% vs. 46.7%; P < 0.0001) (Figure 4D). These studies demonstrate that OSU-CLL is a useful tool for testing CLL therapeutics.

### OSU-CLL Engrafts and Produces a CLL Phenotype in Immunodeficient Mice

**Figure 5:**
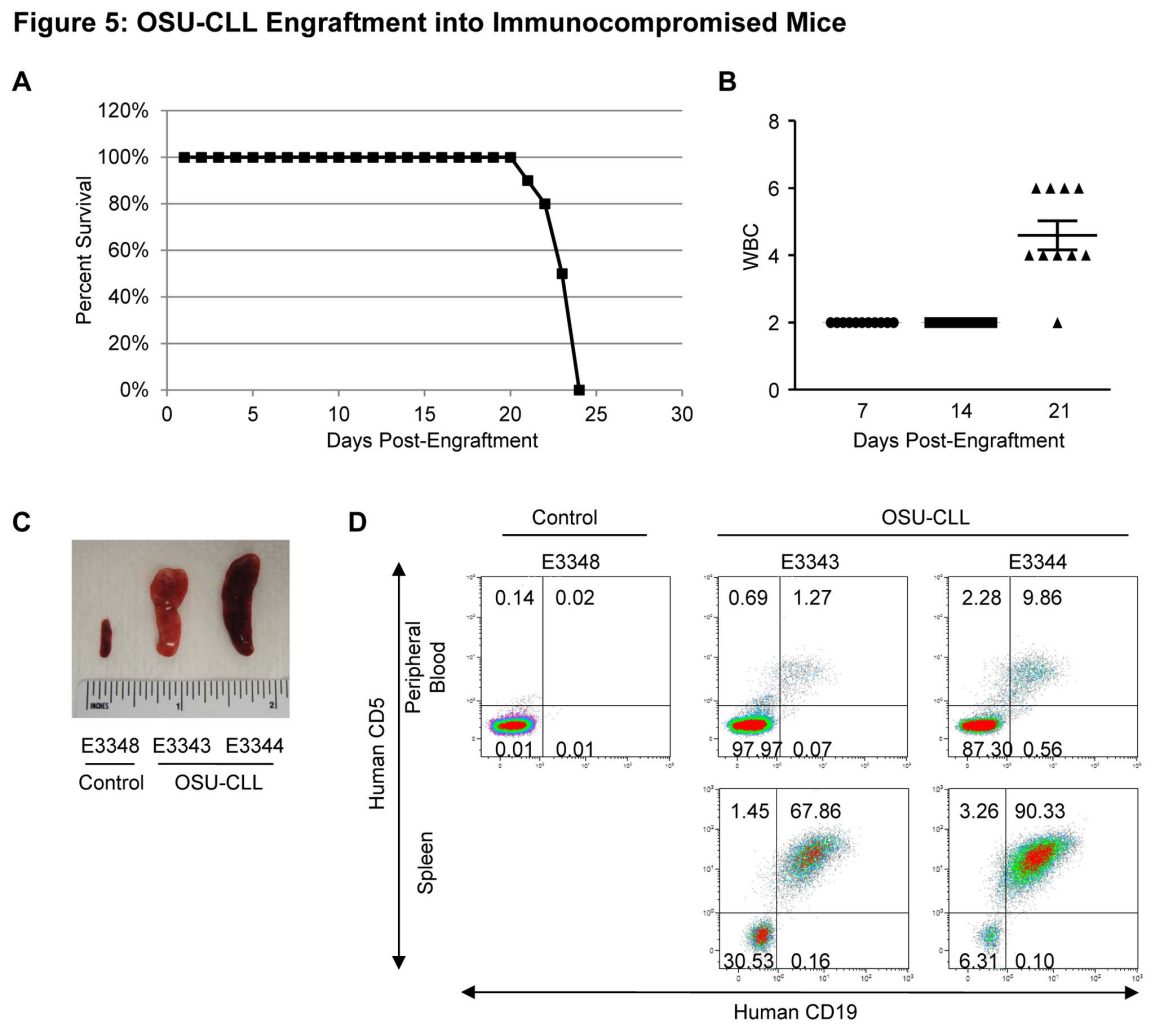
OSU-CLL engrafts into immunodeficient mice. **A**. Survival curve for NOG mice engrafted intravenously with OSU-CLL (1 x 10^7^ cells; N = 10). **B**. White blood cell count (WBC) at 7, 14 and 21 days post-engraftment, determined by Giemsa staining of peripheral blood smears. **C**. Spleens collected at the time of sacrifice from a non-engrafted control animal (left) and two animals engrafted with OSU-CLL (middle, right). **D**. Flow cytometric analysis of surface human CD19 and human CD5 in a non-engrafted control animal and two representative animals engrafted with OSU-CLL. Leukemic cells were detected in both the peripheral blood and spleen cells of the engrafted animals.

Improved animal models to study molecular mechanisms as well as drug efficacy and toxicity *in vivo* are always desirable to further enhance CLL molecular studies. We examined whether OSU-CLL engraftment into mice would produce a phenotype similar to that observed in other CLL xenograft models, and accurately represent human disease. OSU-CLL cells were transplanted into NOG (NOD/Shi-scid/IL-2Rγ^null^) mice, which lack functional B, T and NK cells. This strain was chosen based on prior attempts to transplant EBV-positive B-cells into CB17-SCID animals, possibly due to residual NK cell function which impedes engraftment [39]. A total of 1x10^7^ cells were engrafted by tail vein injection (N = 10), and animals were monitored weekly for increased white blood cell count (WBC) and bi-weekly for human CD19 and CD5 co-expression in PBMCs. The mice exhibit signs of disease (also common in other lymphoblast cell line transplant models) by approximately 3 weeks post-engraftment, and all 10 animals were sacrificed (due to significant hind limb paralysis) between 21-25 days post-engraftment (Figure 5A). However, unlike many other B-cell line transplant models, the onset of disease also was evident by increased WBC count (Figure 5B). Furthermore, these mice developed enlarged spleens (Figure 5C) and flow cytometric analysis of peripheral blood, spleen and bone marrow cells revealed numerous CD19+/CD5+ leukemic cells (Figure 5D, Figure S3), indicating that transplanted cells could migrate to secondary lymphoid organs. Relative to a non-engrafted control, full pathology evaluation of OSU-CLL-bearing mice (N=2) revealed a consistent set of macroscopic and microscopic findings including moderate weight loss (just under 20%) accompanied by lymphocytic leukemia with widespread infiltration of large neoplastic lymphocytes into multiple organs (bone marrow, spleen, liver, kidney, ovaries, epidural space, and pharynx/larynx). These results provide evidence that engrafting OSU-CLL into immunodeficient mice is feasible and that this xenograft model represents an aggressive form of CLL-like disease.

## Discussion

Herein we describe two new cell lines derived from a CLL patient following EBV transformation which bear distinct genetic features atypical of other available lines. A recent review demonstrates that several immortalized cell lines generated from CLL patients in fact were not derived from the CLL clone [15]. We validated through analysis of IGHV rearrangement that not only is OSU-CLL derived from the originating patient’s CLL clone, but the companion OSU-NB originates from the normal B cell population within this patient. These cell lines therefore add to the limited repertoire of CLL lines available for *in vitro* and *in vivo* studies of CLL biology.

The use of transformed cell lines with high EBV levels can introduce several obstacles to *in vitro* studies, including enhanced and constitutive NF-κB activation. However, we have determined that even though OSU-CLL has a high level of basal NF-κB activity, this line still responds to NF-κB activation mediated by CD40L and CpG (Figure S4). Yet expression studies of some genes may be impacted by the EBV. The expression of BCL2, which is regulated by NF-κB, was not detected as differentially expressed between OSU-CLL and OSU-NB even though this gene is significantly over-expressed in primary CLL relative to normal B cells. EBV infection that up-regulates NF-κB in both cell lines may be responsible for the lack of differential BCL2 expression. However many differences between CLL B-cells and normal B-cells are recapitulated in the cell lines, including increased expression of LEF1, ID3 and CD22; and reduced expression of AICDA in OSU-CLL relative to OSU-NB. This indicates that these cell lines are useful tools for studies in CLL gene expression and biology.

OSU-CLL exhibits trisomy 12, which has recently been reported to be associated with the mutation of NOTCH1 [40]. Despite this association in patients, we found that OSU-CLL has wild-type NOTCH1. This discrepancy is important, because mutant NOTCH1 also commonly occurs with unmutated IGHV and high CD38 expression, both characteristics lacking in OSU-CLL. While trisomy 12 is one of the more common chromosomal abnormalities present in CLL, the recent identification of trisomy 12 as a driver mutation suggests that additional studies into the mechanism(s) of disease progression that may be regulated by genes on this chromosome are warranted [27]. The OSU-CLL cell line therefore provides the perfect tool for these studies.

Interestingly, OSU-CLL also has an additional copy of chromosome 19, an abnormality that is relatively rare in CLL. Among the genes present on chromosome 19 are those involved in BCR signaling (CD22), pre-B cell development (LILRA4), and B cell survival and proliferation (TCF3/E2A, and ATF5). CD22 is of particular interest as it is a target approved for monoclonal antibody therapy in several B-cell leukemias and lymphomas. Knock-down of TCF3/E2A in CLL cells causes an increase in spontaneous apoptosis and decreased expression of cell survival genes (p21, p27 and Mcl-1) [41]. Similarly, ATF5 is involved in cell cycle progression and apoptosis, and has been shown to be over-expressed in CLL with 11q deletions or trisomy 12 versus other cytogenetic sub-groups and associated with shorter time to treatment [42]. The significance of these genes present on chromosome 19 to CLL cell transformation deserves more study.

The OSU-CLL line has migratory properties similar to primary CLL cells, migrating more readily toward CXCL12 compared to OSU-NB. This difference could be due in part to the increased expression of CXCR4 in the OSU-CLL. However OSU-CLL exhibits enhanced migration relative to OSU-NB even independently of chemokine, indicating the involvement of other factors, such as enhanced BCR signaling. While the migration of OSU-CLL is similar to that described for other B cell lines, the ability of OSU-CLL to generate substantial peripheral disease makes this line more attractive as a xenograft model, as it would allow *in vivo* studies of CLL cell migration. Aggressive expansion similar to OSU-CLL has been described previously only with serial transplantation of spleen cells from a TCL1-transgenic mouse [43].

One limitation of pharmacologic studies with primary CLL cells is the inability to stably transfect genes to study their function and interactions. We found that Zap70 expression was evident in early passage OSU-CLL, but decreased over time in culture (Figure S5A), providing a nice system to over-express Zap70 for functional studies. We have successfully transduced Zap70 into OSU-CLL via retroviral infection using both a constitutive and a tetracycline-inducible vector (Figure S5B). The ability to easily transduce OSU-CLL allows interrogation of gene function for molecular studies, and demonstrates the potential manipulation of OSU-CLL to study function of genes relevant to CLL biology. Additionally, our data demonstrate that OSU-CLL responds to therapeutics approved for clinical treatment of CLL, including antibody therapies. Therefore this cell line provides a new model for pre-clinical testing of new agents, as well as studies in drug resistance due to oncogene over-expression.

Finally, while spontaneous models of B-cell malignancy (such as the TCL1 mouse) are useful tools, they carry certain disadvantages such as the inability to evaluate anti-human therapeutic antibodies which are usually not cross-reactive with the equivalent mouse protein. Therefore having a CLL-derived cell line for use in xenograft studies could be particularly beneficial for this purpose. Several CLL xenograft models have been described, however many of these models use cell lines with an atypical CLL phenotype (i.e. lacking CD5 expression), and exhibit either minimal or no peripheral disease [44–47]. These studies indicate the necessity for the development of novel xenograft models for the study of this disease. We demonstrate that OSU-CLL is able to engraft into immunodeficient mice, and in this setting produces a phenotype that facilitates monitoring disease in different compartments (blood, bone marrow, and spleen; Figure 5 and Figure S3). The localization to the secondary lymphoid organs provides a xenograft model that will allow for studies investigating CLL B cell migration, and possibly lymphocytosis which is observed in response to many CLL therapeutic agents.

## Supporting Information

Figure S1 **Morphology and viral protein expression in EBV transformed cell lines.** A. Phase contrast images (10X resolution, inset at 20X) of OSU-NB (left) and OSU-CLL (right). B. Immunoblot analysis for EBV proteins (LMP1, EBNA2, EBNA3a and BXLF1) in the OSU-NB, and OSU-CLL cell line at various times in culture. Blots are probed with actin as a loading control. Results shown are representative of 3 individual experiments.
(TIF)

Figure S2 **Chromosome specific gene expression in the OSU-CLL and OSU-NB cell lines.** The total number of genes and the percent (based on the total number of genes on the indicated chromosome) is shown for chromosomes 12 and 19. The same analysis is shown for chromosomes 1 and 2 for comparison purposes.
(TIF)

Figure S3 **Flow cytometric analysis in spleen and bone marrow of engrafted animals.** Flow cytometric analysis of surface human CD19 and human CD5 in peripheral blood, spleens and bone marrow in additional animals engrafted with OSU-CLL.
(TIF)

Figure S4 **NF-κB activation in the OSU-CLL and OSU-NB cell lines in response to cell stimulation.** OSU-NB and OSU-CLL cell line (passage 25) were treated with 1.7 µM CpG for 3 hours or 500 ng/mL recombinant CD40L for 1 hour. Nuclear and cytosolic lysates were prepared and immunoblot analysis was performed for NF-κB proteins (RelB and p65). Blots are probed with Brg1 and Tubulin as controls for the nuclear and cytosolic isolation. Results shown are representative of 3 individual experiments.
(TIF)

Figure S5 **Zap70 protein expression and retroviral infection in OSU-CLL.** **A**. Immunoblot analysis for Zap70 protein in OSU-NB, and OSU-CLL cell line at various times in culture. Blots are probed with actin as a loading control. Results shown are representative of 3 individual experiments. **B**. OSU-CLL cells were stably transduced with both a constitutive (left) and a doxycycline inducible (right) expression construct for Zap70. In the inducible cell line, immunoblot analysis was performed for Zap70 protein after 48 hours with and without induction with 500 ng/mL doxycycline.
(TIF)

Table S1 **Top 50 Up-Regulated Genes in OSU-CLL versus OSU-NB.** Gene expression analysis results from Affymetrix U133 microarray for the OSU-NB and OSU-CLL cell lines, analyzed at passage 25.
(TIF)

Table S2 **Top 50 Down-Regulated Genes in OSU-CLL versus OSU-NB.** Gene expression analysis results from Affymetrix U133 microarray for the OSU-NB and OSU-CLL cell lines, analyzed at passage 25.
(TIF)
