# Supplementary figures and images for: Characterization of a New Chronic Lymphocytic Leukemia Cell Line for Mechanistic In Vitro and In Vivo Studies Relevant to Disease
Source: PLoS One. 2013 Oct 9;8(10):e76607. doi: 10.1371/journal.pone.0076607 (PMC3793922; doi:10.1371/journal.pone.0076607)

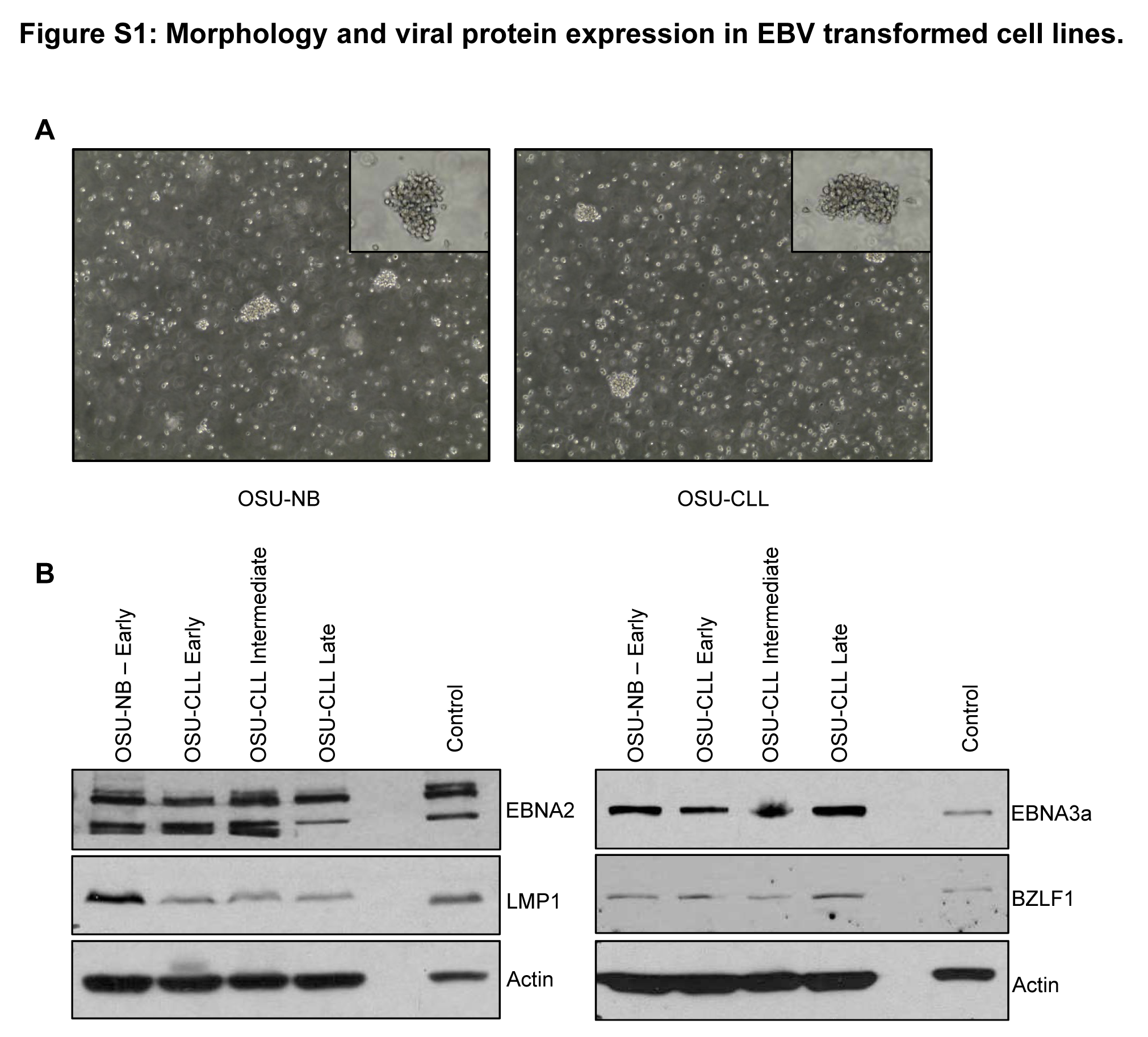

Supplement: Figure S1 — Morphology and viral protein expression in EBV transformed cell lines. A. Phase contrast images (10X resolution, inset at 20X) of OSU-NB (left) and OSU-CLL (right). B. Immunoblot analysis for EBV proteins (LMP1, EBNA2, EBNA3a and BXLF1) in the OSU-NB, and OSU-CLL cell line at various times in culture. Blots are probed with actin as a loading control. Results shown are representative of 3 individual experiments. (TIF) [file pone.0076607.s001.tif]

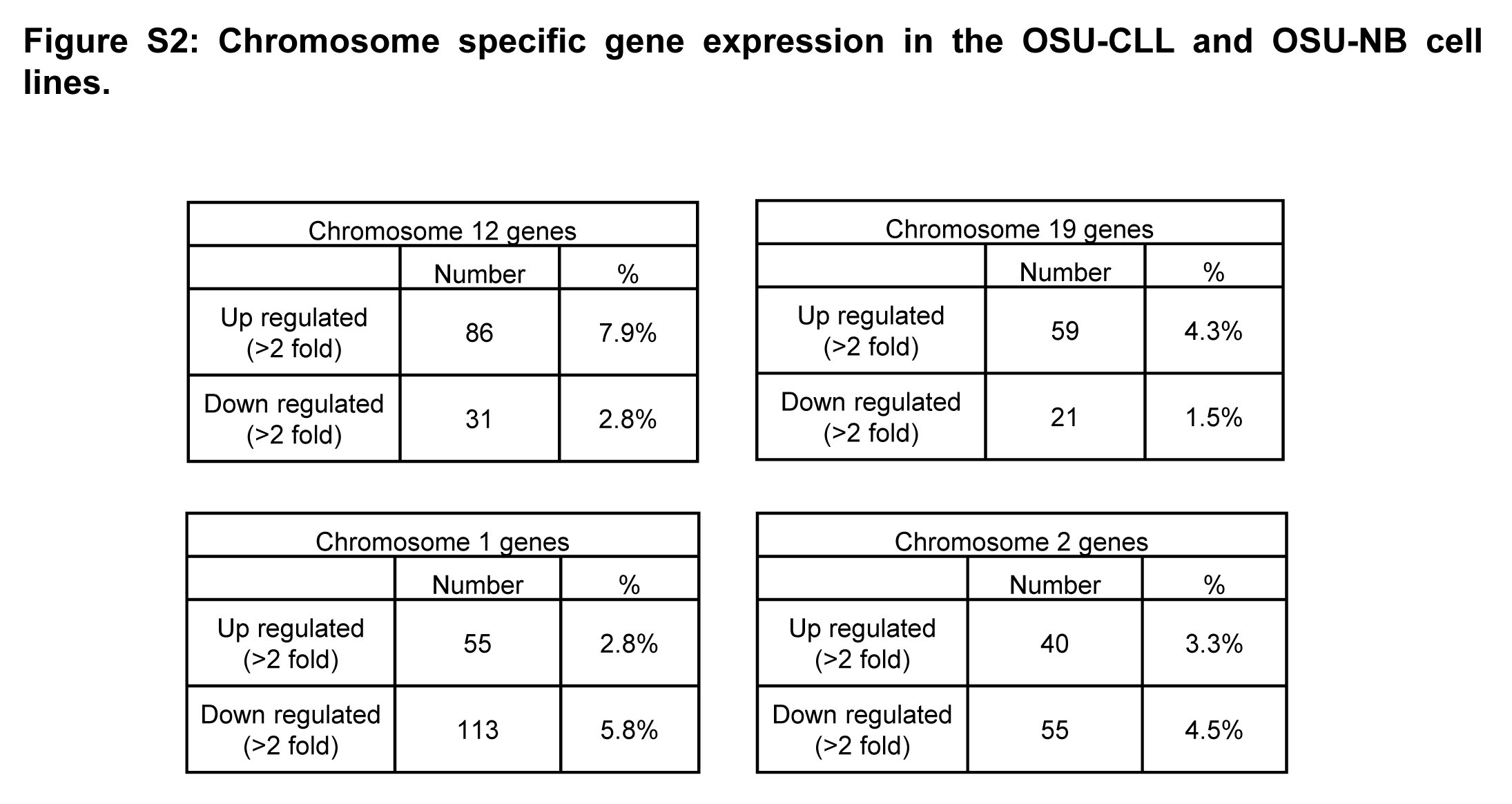

Supplement: Figure S2 — Chromosome specific gene expression in the OSU-CLL and OSU-NB cell lines. The total number of genes and the percent (based on the total number of genes on the indicated chromosome) is shown for chromosomes 12 and 19. The same analysis is shown for chromosomes 1 and 2 for comparison purposes. (TIF) [file pone.0076607.s002.tif]

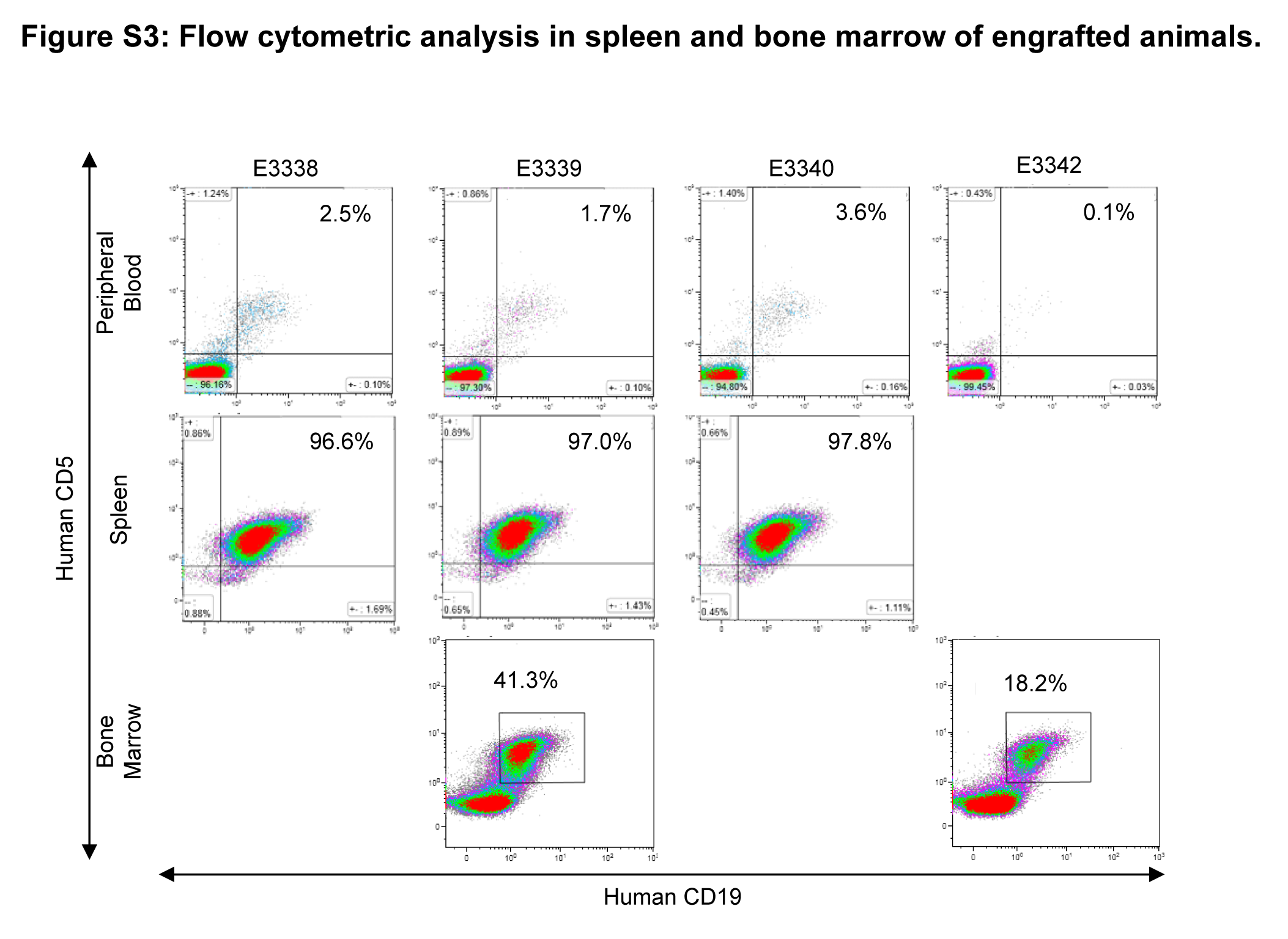

Supplement: Figure S3 — Flow cytometric analysis in spleen and bone marrow of engrafted animals. Flow cytometric analysis of surface human CD19 and human CD5 in peripheral blood, spleens and bone marrow in additional animals engrafted with OSU-CLL. (TIF) [file pone.0076607.s003.tif]

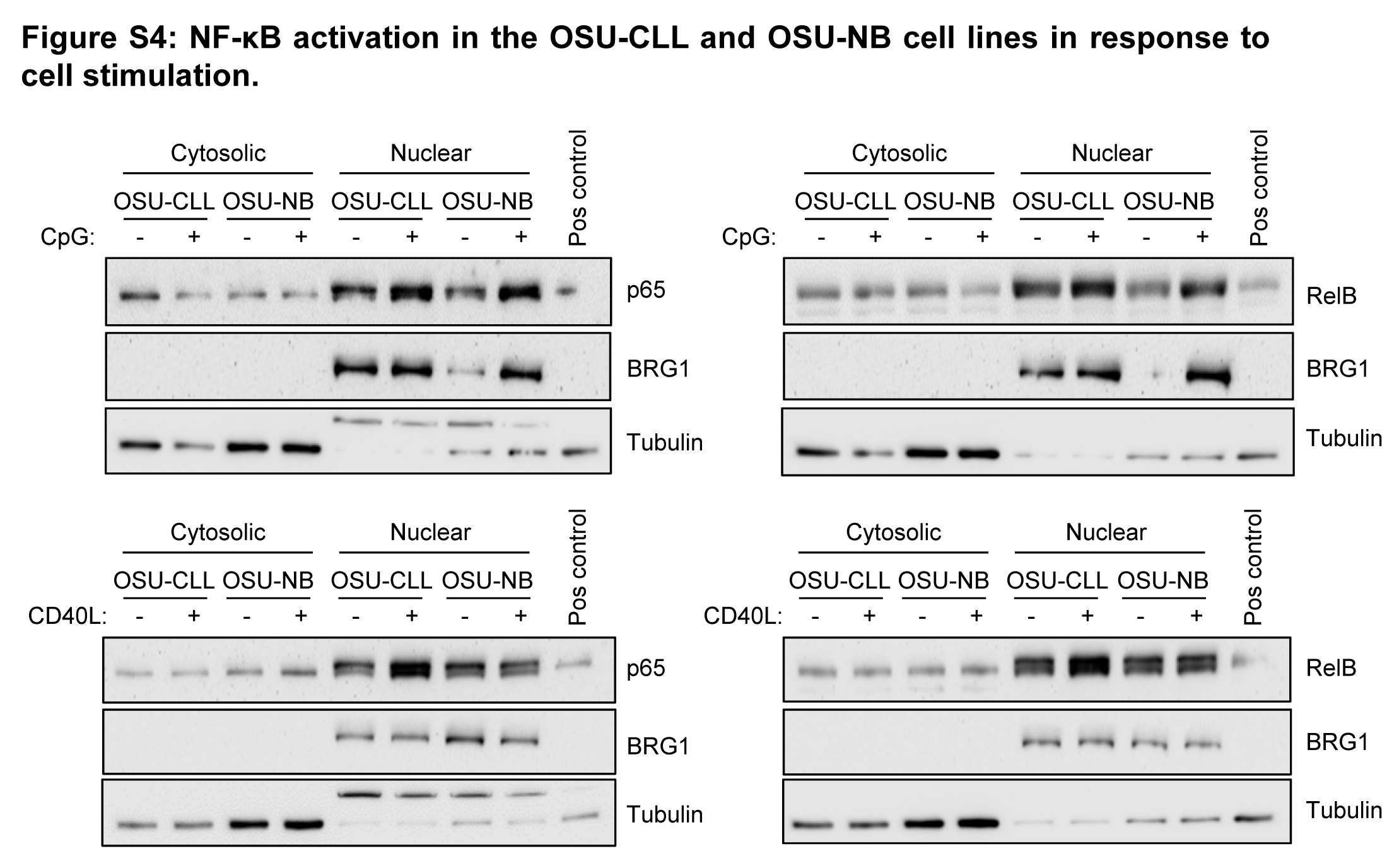

Supplement: Figure S4 — NF-κB activation in the OSU-CLL and OSU-NB cell lines in response to cell stimulation. OSU-NB and OSU-CLL cell line (passage 25) were treated with 1.7 µM CpG for 3 hours or 500 ng/mL recombinant CD40L for 1 hour. Nuclear and cytosolic lysates were prepared and immunoblot analysis was performed for NF-κB proteins (RelB and p65). Blots are probed with Brg1 and Tubulin as controls for the nuclear and cytosolic isolation. Results shown are representative of 3 individual experiments. (TIF) [file pone.0076607.s004.tif]

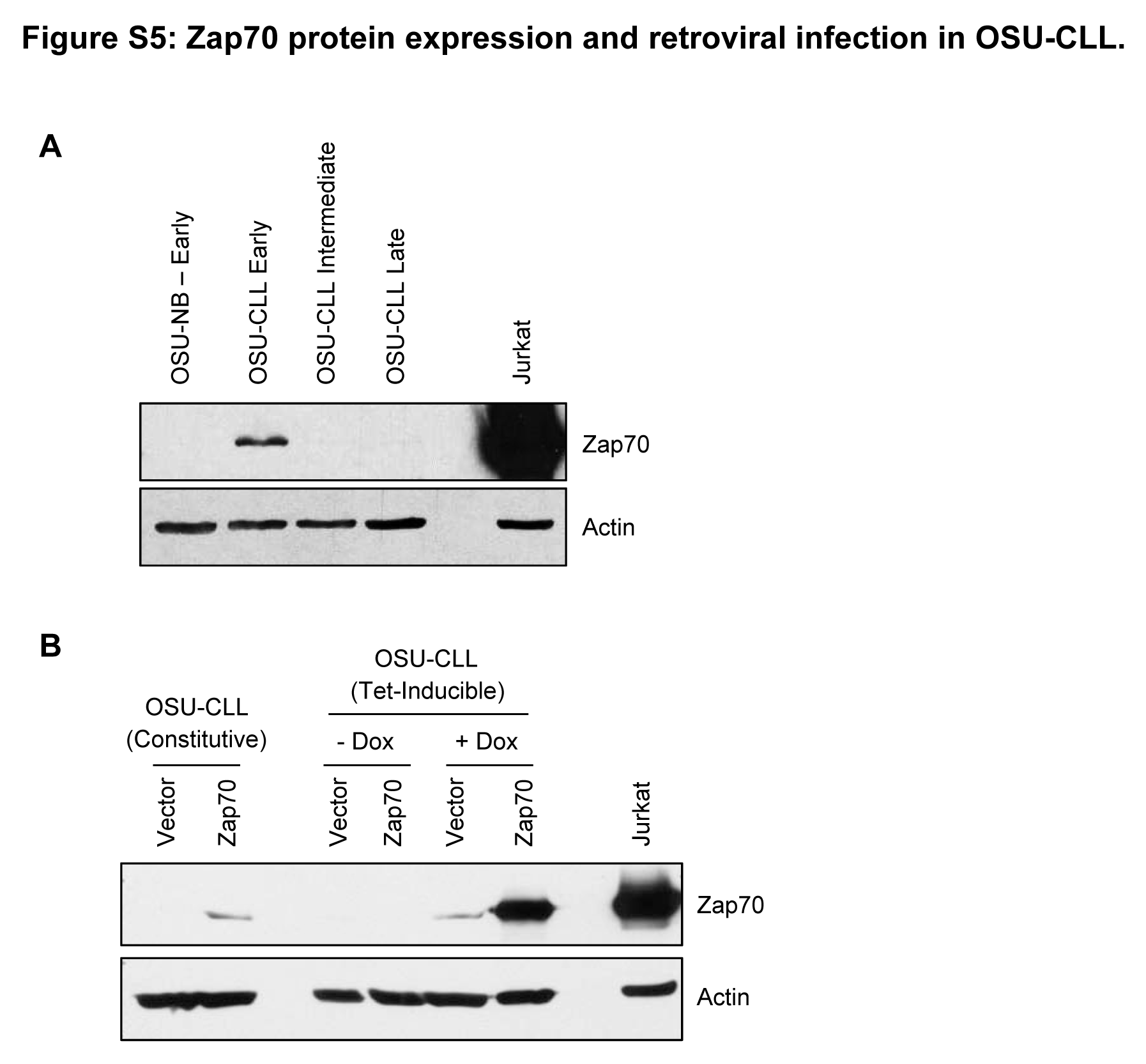

Supplement: Figure S5 — Zap70 protein expression and retroviral infection in OSU-CLL. A. Immunoblot analysis for Zap70 protein in OSU-NB, and OSU-CLL cell line at various times in culture. Blots are probed with actin as a loading control. Results shown are representative of 3 individual experiments. B. OSU-CLL cells were stably transduced with both a constitutive (left) and a doxycycline inducible (right) expression construct for Zap70. In the inducible cell line, immunoblot analysis was performed for Zap70 protein after 48 hours with and without induction with 500 ng/mL doxycycline. (TIF) [file pone.0076607.s005.tif]

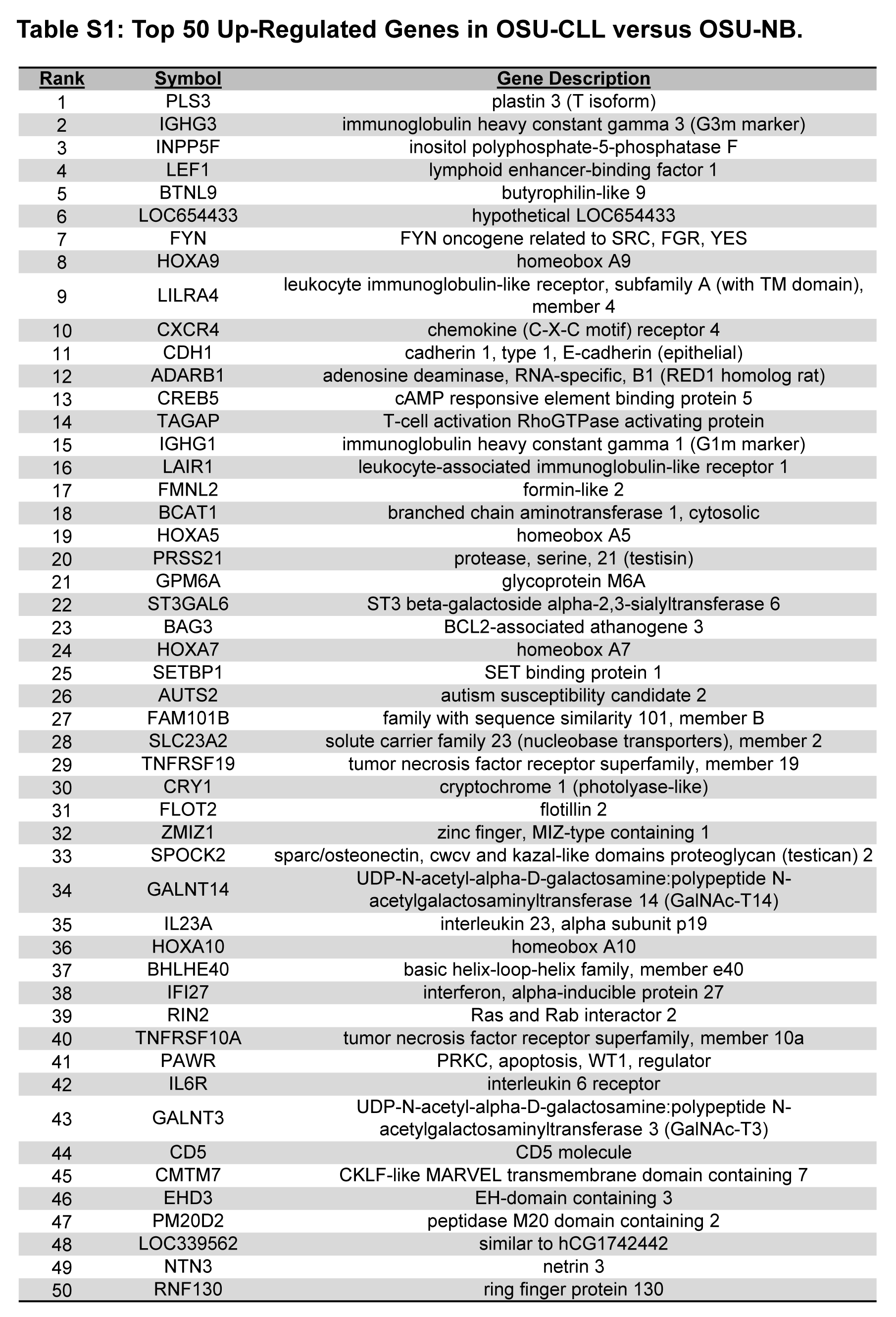

Supplement: Table S1 — Top 50 Up-Regulated Genes in OSU-CLL versus OSU-NB. Gene expression analysis results from Affymetrix U133 microarray for the OSU-NB and OSU-CLL cell lines, analyzed at passage 25. (TIF) [file pone.0076607.s006.tif]

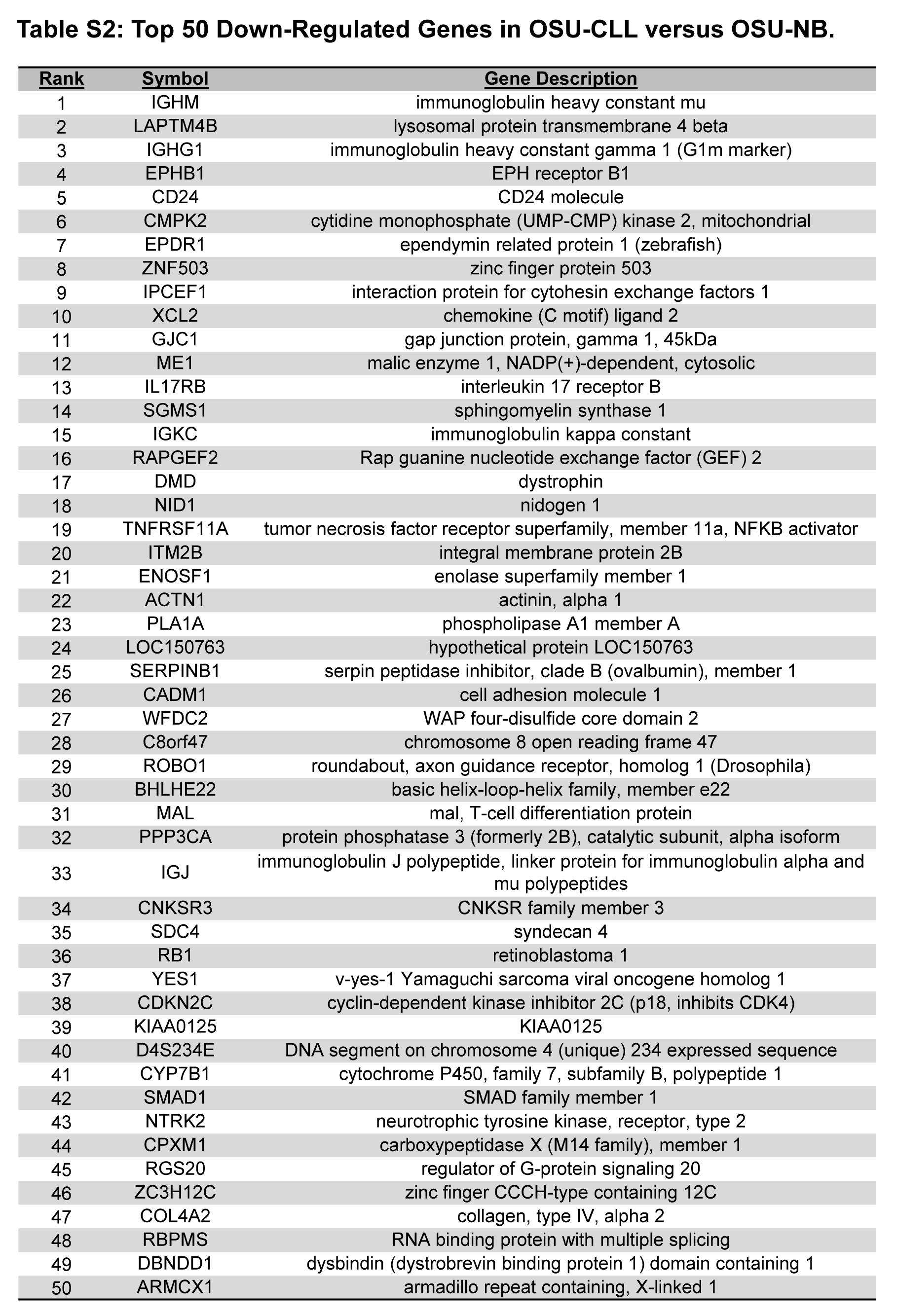

Supplement: Table S2 — Top 50 Down-Regulated Genes in OSU-CLL versus OSU-NB. Gene expression analysis results from Affymetrix U133 microarray for the OSU-NB and OSU-CLL cell lines, analyzed at passage 25. (TIF) [file pone.0076607.s007.tif]
